# Cognitive Remediation for Psychosis in Virtual Reality (ThinkTactic VR): Qualitative, Iterative, and User-Centered Codevelopment Study

**DOI:** 10.2196/69359

**Published:** 2025-07-11

**Authors:** Jasmin Yee, Hannah Matheson, Bryce J M Bogie, Émilie Du Perron, Alexandra Thérond, Maëlle Charest, Catheleine van Driel, Marika Goyette, Ya Ting Lei, Chelsea Noël, Kagusthan Ariaratnam, Greg Collins, Chris Gorman, Ana-Maria Cretu, Simon Tremblay, Marie-Christine Rivard, Catherine Cullwick, Crystal Morris, David G Attwood, Alexandra Baines, Angela Stewart, Stéphane Bouchard, Christopher R Bowie, Synthia Guimond

**Affiliations:** 1 University of Ottawa Institute of Mental Health Research at The Royal Royal Ottawa Health Care Group Ottawa, ON Canada; 2 Department of Psychology Carleton University Ottawa, ON Canada; 3 Department of Occupational Science & Occupational Therapy University of Toronto Toronto, ON Canada; 4 Department of Cellular and Molecular Medicine University of Ottawa Ottawa, ON Canada; 5 Département de psychologie Université du Québec à Montréal Montreal, QC Canada; 6 Département de psychoéducation et de psychologie Université du Québec en Outaouais Gatineau, QC Canada; 7 Department of Psychiatry University Medical Center Groningen Groningen The Netherlands; 8 Department of Psychology Lakehead University Thunder Bay, ON Canada; 9 Département d’informatique et d’ingénierie Université du Québec en Outaouais Gatineau, QC Canada; 10 Professional Practice Royal Ottawa Mental Health Centre Royal Ottawa Health Care Group Ottawa, ON Canada; 11 Department of Mental Health The Ottawa Hospital Ottawa, ON Canada; 12 Department of Psychiatry University of Ottawa Institute of Mental Health Research at The Royal Ottawa, ON Canada; 13 Department of Psychology Queen's University Kingston, ON Canada

**Keywords:** virtual reality, cognitive remediation, user-centered approach, neurocognition, social cognition, community functioning

## Abstract

**Background:**

Cognitive remediation improves cognition and psychosocial functioning in individuals with psychotic disorders. The use of virtual reality (VR) to deliver cognitive remediation in immersive environments that mimic real cognitively challenging situations has the potential to increase engagement to treatment and further enhance its impact on functioning.

**Objective:**

We aimed to codevelop a cognitive remediation program in VR with individuals with psychotic disorders and health care professionals to identify and address their needs.

**Methods:**

Individuals with lived experience of a psychosis-spectrum condition (n=11) met 9 times and the health care professionals (n=7) met 3 times. Participants discussed personal and professional opinions on the challenges associated with cognitive difficulties in individuals with psychotic disorders. They also provided feedback on the program development.

**Results:**

We discerned 4 themes from the content expert working groups: the need for a program to address cognitive impairments, the key program design elements to support cognitive rehabilitation, the importance of leveraging technology as an intervention tool, and the need to improve community functioning. In total, 3 themes were identified for the health care professionals: the need for a clinically relevant program that addresses the research-to-practice gap, the need to improve patient engagement in services, and the need for a program that addresses the limited resources in health care. The needs of our end-user experts were placed at the center of the program development process. When possible, we also integrated their suggestions, like the incorporation of a virtual coach within the immersive environment.

**Conclusions:**

Individuals with lived experience and health care professionals have distinct needs, which have informed the co-design of a novel cognitive remediation program in VR, ThinkTactic VR. To our knowledge, ThinkTactic VR is one of the first co-designed and codeveloped cognitive remediation programs in VR using an iterative, user-centered approach involving both individuals with psychotic disorders and health care professionals.

## Introduction

### Background

Psychotic disorders, such as schizophrenia and schizoaffective disorder, are characterized by positive and negative symptoms, as well as cognitive impairments [1]. Neurocognitive impairments (eg, attention and memory) and social-cognitive impairments (eg, emotion regulation and theory of mind) are common in individuals with psychotic disorders [2-4]. These impairments are associated with important challenges in community functioning and in performing daily activities [5,6]. Although current pharmacological approaches successfully reduce positive symptoms, they do not have a consistent positive impact on cognitive functions [7]. This limitation has encouraged the development of nonpharmacological interventions specifically targeting cognitive impairments in this population [8].

Cognitive remediation (CR) is a targeted approach that seeks to improve cognitive deficits and community functioning through cognitive exercises, strategies, and rehabilitative practices [9-12]. While the delivery method of CR differs, 4 core features of CR have been identified as follows: (1) the presence of exercises to improve cognition, (2) the presence of a trained therapist, (3) the identification and development of cognitive strategies, and (4) support for the transfer of skills to community functioning [9]. Studies have consistently provided evidence for the efficacy of CR in improving neurocognition and social cognition in psychotic disorders [12-14]. Despite increasing supporting evidence of CR, the transfer of learned skills and generalizability of skills to psychosocial functioning is more limited [12,15,16]. Furthermore, CR programs are sometimes accompanied by diminished motivation to continue with treatment, resulting in high dropout rates [16-18]. To address these limitations, one promising avenue is leveraging virtual reality (VR) to deliver CR interventions [19].

### The Use of VR as a Rehabilitation Tool

VR is a computer-generated technology that creates 3D environments reminiscent of real-world scenarios, thus allowing for the presentation of more ecological stimuli [1,20,21]. The resulting increase in ecological validity provides a more realistic environment to learn and practice cognitive skills compared to traditional, computer-based CR modules [21]. VR is a powerful clinical tool that allows for the creation of experiences (eg, public transportation and kitchen-based scenarios) that are difficult to replicate in a treatment setting [22-24]. In addition, the immersive aspect of VR programs can provoke cognitive and emotional responses similar to those experienced in real-world settings [25,26]. Previous research has identified VR as a safe and effective method for individuals with psychotic disorders [19,27-29]. Cognitive programs in VR have shown promising results to improve neurocognition and social cognition [19,28]. However, these results are often based on small samples and pilot studies [30].

To date, there are a limited amount of CR programs in VR available for individuals living with psychotic disorders [30], including interventions that target both neurocognition and social cognition. Moreover, most CR programs in VR have been developed with minimal direct input from end users [22,31,32]. The few programs that have incorporated user feedback have done so to a limited extent, highlighting an area for potential improvement in the design and efficacy of these interventions [32]. A lack of user engagement during program development is one potential reason for program implementation failure [22]. As such, there is a need to codevelop a CR program in VR for individuals with psychotic disorders. Codeveloping a CR program in VR that includes scenarios that apply to real life and incorporates the key 4 elements of CR can address some of the current limitations commonly found in CR studies and can furthermore facilitate the transfer to community functioning [33].

### Involving a Multidisciplinary Team in Program Design

To effectively codevelop a CR program in VR, Birckhead et al [22] recommend that VR applications be co-designed with direct input from individuals who would use and benefit from these programs. Current research has indicated that incorporating ideas from individuals with lived experience of a psychosis-spectrum condition (ie, content experts [CEs]) is integral for their symptom management and can improve their treatment motivation and outcomes [32,34]. It is also vital to include input from health care professionals (HCPs) in treatment design, given their expertise in providing care and understanding of psychosis-related symptomology and its impact on clinical and functional outcomes [22]. HCPs can also identify potential facilitators and barriers to the clinical implementation of such treatment programs. Including CEs and HCPs in the codevelopment process could improve engagement and adherence, as well as the content relevance of the program.

### Aims and Objectives

The primary objective of this study is to report on the cocreation of a CR program in VR involving both CEs and HCPs. To achieve this, we identified and addressed the specific treatment needs of these 2 distinct end user groups. We adopted an iterative development process, ensuring the VR program effectively met their needs. This study outlines the codevelopment process, from the initial concept to the creation of the finalized program.

## Methods

### Study Design

We followed 2 established frameworks: the recommendations for developing effective VR interventions [22] and the 4 core features recommended for CR programs [9]. During the study, working groups were conducted separately for CEs and HCPs, during which participants discussed their needs and provided feedback on the VR program. Inductive thematic analyses were then performed for each group, and the feedback was incorporated into the development of the program after each session. This iterative process ensured that the evolving program effectively addressed the needs of both user groups.

### Ethical Considerations

This study received ethical approval from the Royal Ottawa Health Care Group Research Ethics Board (REB 2019016) and the University of Québec in Outaouais (REB 2022-1869). All participants provided written informed consent at the beginning of the study and verbal consent to continue participating was obtained before every subsequent working group. Participant data was de-identified and stored separately from consent forms. Participants received CAD $20 (approximately US $12) in cash or a gift certificate for each working group session they attended.

### Participants

#### CE Groups

In total, 11 CEs were recruited from the Royal Ottawa Mental Health Centre (ROMHC) and the local community in Ottawa, Ontario. Participants were referred to the study by their clinician or self-referred after viewing flyers and brochures distributed in Ottawa. Of the 11 CEs, 5 (45%) withdrew from the study before the 3 years of development ended. Reasons for withdrawal included loss of interest (1/11, 9%) and time constraints (1/11, 9%). The 3 other participants did not provide a reason for their withdrawal. Demographic information about the individuals included in our CEs group can be found in Multimedia Appendix 1.

Inclusion criteria were (1) aged ≥18 years, (2) ability to read and speak fluent English, (3) diagnosed with a psychotic disorder, confirmed by the referring clinician or review of electronic medical records after consent, and (4) were on a stable medication regimen for at least 1 month before enrollment. Potential CEs were excluded if they (1) had vision problems without contact lenses or glasses that fit with the VR headset, (2) had neurological or medical disorders (other than a psychotic disorder) that could contribute to cognitive impairment, (3) had epilepsy or had a history of seizures, (4) had a recent history of substance abuse or dependence (within the past 3 months), (5) had a previous history of motion sickness or cybersickness (ie, motion sickness related to virtual immersion), or (6) had decisional incapacity requiring a guardian.

#### HCP Group

In total, 7 HCPs consisting of psychiatrists (2/7, 29%), clinical psychologists (2/7, 29%), a neuropsychologist (1/7, 14%), and occupational therapists (OTs; 2/7, 29%) were recruited from the ROMHC and collaborative institutions. In addition, 2 collaborators from other institutions were invited to participate in the working groups due to their expertise in CR and VR psychological interventions. No HCPs withdrew from the study.

HCPs meeting inclusion criteria were (1) an HCP and (2) had 2 or more years of clinical experience with individuals with psychotic disorders or with VR interventions to ensure they had the necessary background experience. The same exclusion criteria for CEs were also applied for HCPs.

### Procedure

#### CE Working Groups

**Figure 1:**
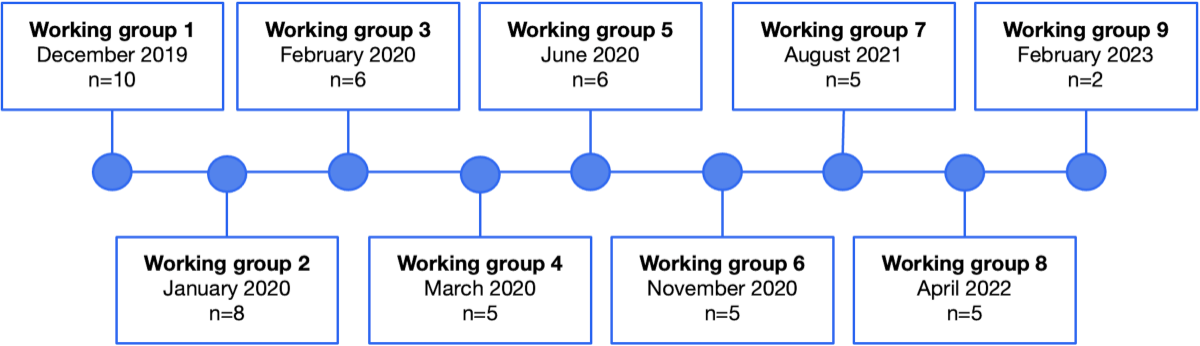
Content expert working group timeline (n=11).

CEs were invited to in-person or web-based (Zoom Communications, Inc) meetings for an enrollment visit and 9 working group sessions (Figure 1). The same participants were invited to each working group session. CE working group sessions 1 to 5 were completed in person at the ROMHC, while the rest of the sessions were facilitated through Zoom for Healthcare due to the COVID-19 pandemic. At the initial enrollment visits, CEs were individually interviewed by a trained research assistant. During this visit, a detailed description of the study and their role and responsibilities as research partners were discussed and informed consent was obtained. In addition, initial testing of a VR environment prototype was completed. When participants completed initial prototype testing during their enrollment visit, the Simulator Sickness Questionnaire was administered to assess cybersickness (Kennedy et al [35]; Multimedia Appendix 1). Afterward, a discussion was initiated with the CEs to explore their needs and gather their initial reactions to the program. These activities established rapport and provided the necessary background information for the first working group.

In the initial 3 working groups, 2 to 3 facilitators (SG, HM, and AT) provided psychoeducation on neurocognition (working group 1), social cognition (working group 2), and community functioning (working group 3). After presenting the topic, the facilitators led a semistructured discussion to gather information from CEs about their personal experiences and opinions on the challenges associated with psychotic disorders as they related to the topic. These working groups were used to determine the needs of the CEs and the types of VR tasks to include in the training program. The remaining 6 working groups were facilitated by 2 study personnel (HM, AT, CN, or JY) who focused on gathering feedback on prototype versions of the program from CEs.

#### HCP Working Group

**Figure 2:**
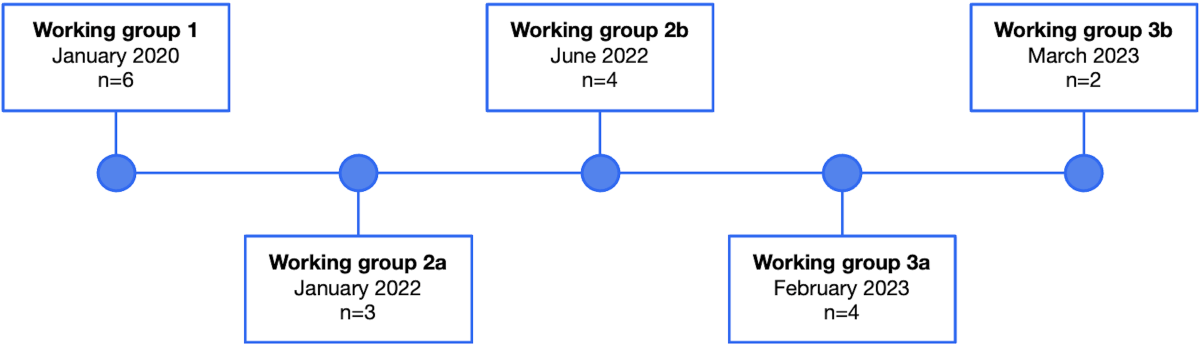
Health care professional working group timeline (n=7). The second working group was split into 2 separate sessions (group 2a and group 2b), along with the third working group (group 3a and group 3b) to accommodate participant availability.

The 7 HCPs were invited to 3 working groups between January 2020 and March 2023 (Figure 2). In each working group, 2 facilitators (SG, TR, or JY) facilitated a discussion where HCPs shared their personal opinions on the challenges associated with living with a psychotic disorder and provided program feedback. The first working group was held in person at the ROMHC, with 2 participants joining virtually through Zoom. Due to the COVID-19 pandemic, the remaining working groups were facilitated virtually through Zoom.

In the first working group, informed consent was obtained from HCPs, who then discussed their professional opinions on the treatment challenges associated with psychotic disorders. Afterward, HCPs tested an initial prototype of the VR program. In the remaining 2 working groups, HCPs provided feedback on the other versions of the VR program.

### VR Design

#### User-Centered Iterative VR Program Development

**Figure 3:**
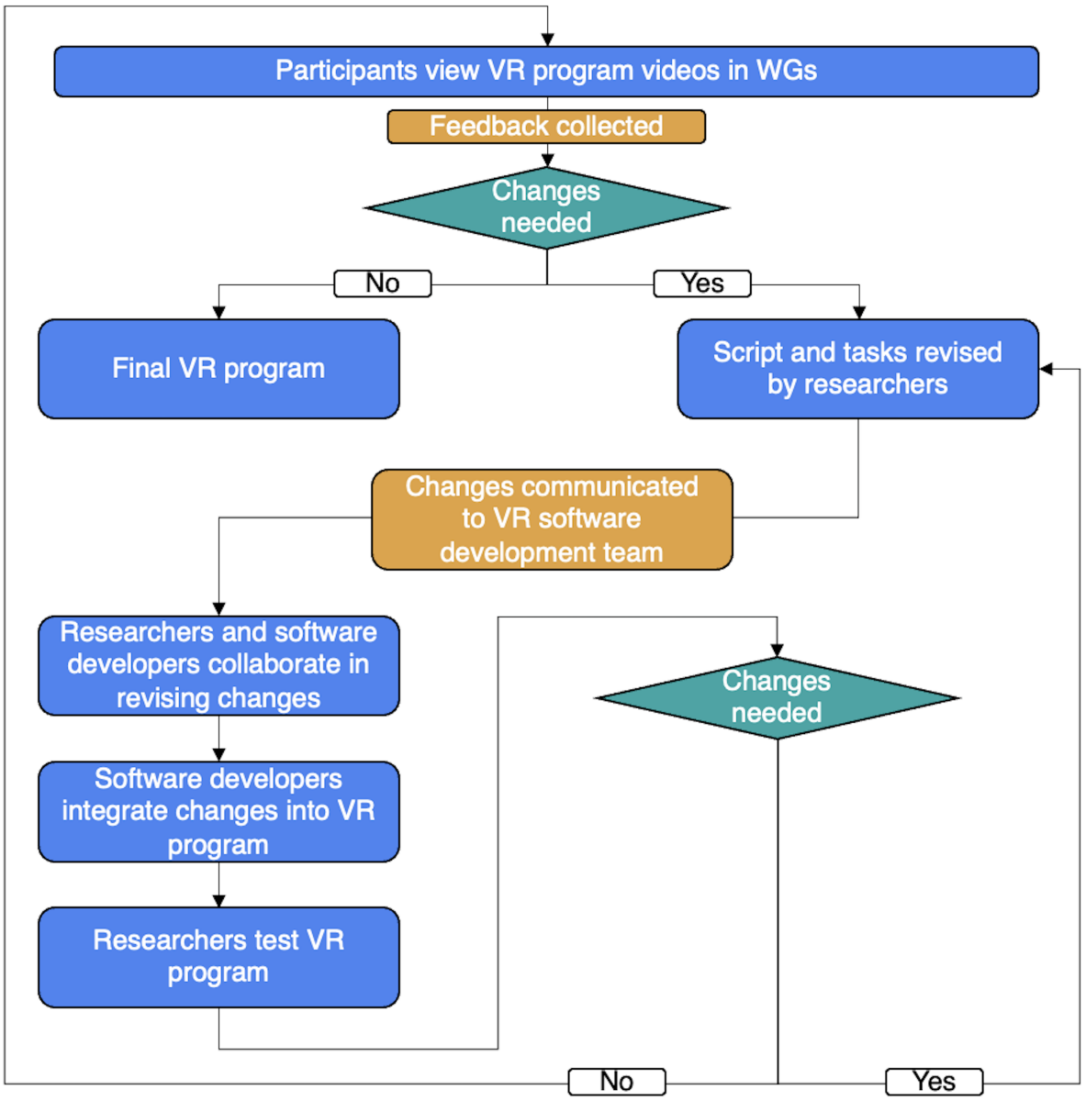
Iterative process to program development. VR: virtual reality; WG: working group.

The VR environments and tasks were developed following a user-centered design process and an iterative approach wherein the perspectives of CEs and HCPs were integrated into all stages of the design process (Figure 3). At the end of the third working group, CEs brainstormed and identified potential VR environments and tasks. On the basis of the feedback collected, examples of VR environments and tasks were showcased in the fourth working group. An iterative process subsequently followed where feedback was collected from CEs, which were later reviewed by the research team. When creating the VR environment, designs had to reflect the needs of CEs and HCPs while adhering to the psychological principles of CR therapy. In collaboration with software developers, the proposed changes also had to abide by current technological parameters and VR design considerations (eg, reducing the incidence of cybersickness). Research staff and software developers continuously tested and provided feedback on these changes. Once the research staff and software developers finalized the changes, the modified CR program in VR was showcased at the subsequent working group, and new feedback was collected.

In addition, a VR coach was integrated into the VR program, providing VR navigation assistance (eg, instructions on how to respond to a question or interact with objects in the environment), feedback on task performance, and CR strategies for a user. The CEs initiated the development of the VR coach, and feedback was continuously provided during its development in the working groups. Research staff collaborated with software developers to create and modify the VR coach accordingly.

#### Software Development

Software development was carried out by the Cyberpsychology Laboratory at the University of Québec in Outaouais, led by SB and MCR. Their team was composed of software developers who specialized in rehabilitative VR environments. Character animations were created based on previous VR simulations produced in the Cyberpsychology Lab, and the VR environments were also partly created using preexisting environment design.

### CR Therapy

The tasks within the VR modules were created by integrating and adapting strategies from CR manuals developed by Bellack et al [36], Friedberg et al [37], and Wykes and Reeder [38], and input from CEs and HCPs. Key strategies for treating social cognition, neurocognition, and community functioning in schizophrenia were identified from the above manuals. The scenario scripts of the VR program development began after the fifth working group session with CEs. Initial drafts were completed through weekly meetings between HM and AT during which feedback from the working group sessions and CR manuals were used to help construct the environment. The CR program in VR was revised after the ninth working group to refine the CR aspect further (JY, SG, BJMB, and CvD).

A similar approach was used to identify the CR strategies that the VR coach in the immersive environment should provide when an individual is having difficulties with a task in the VR program. Additionally, this approach was used to support trained therapists in delivering the CR in VR program sessions.

### Qualitative Data Analysis

A preliminary inductive analysis following the Braun and Clarke [39] model was conducted in March 2021 to identify and subsequently integrate central elements into the program using transcripts from the first 7 CE and the first HCP working groups (HM and CN). Working groups were recorded and transcribed verbatim through Dragon Professional Individual (version 15; HM), with syntactical corrections and identifiable information redacted to safeguard participant anonymity (eg, names of participants, occupation, and work location). To mitigate bias, a team of research staff not involved in the preliminary analysis conducted the final inductive thematic analyses to identify the treatment needs of CEs and HCPs. Recordings of the remaining working groups were transcribed and reviewed to redact identifiable information (MC). Once the transcripts were finalized, the audio recordings were deleted.

Two separate inductive thematic analyses were conducted with CEs and HCPs to acknowledge and isolate the different needs of the 2 participant groups. NVivo (version 12.0, Lumivero) was used to facilitate familiarization, coding, and data organization. The CE analysis was conducted from December 2022 to May 2023 and in September 2023 as working group 9 served as a debrief and presentation of the final VR program. Thematic coding was conducted independently (JY and EDP) for the first 3 working groups. Once no new codes emerged from the text, coders met to create an overall codebook to ensure text comprehension and consensus. The remaining 6 working group transcripts were then equally distributed between the 2 coders, who both used the final codebook to code the remaining transcripts. Themes were generated based on the final codes. With the assistance of external evaluators (CvD, BJMB, AT, and SG), the themes were refined by combining similar themes. Subsequently, subthemes were identified. Once themes were refined and finalized, their essence was defined. The same process was completed for the HCP thematic analysis conducted between February 2023 and May 2023.

## Results

### Overview

**Figure 4:**
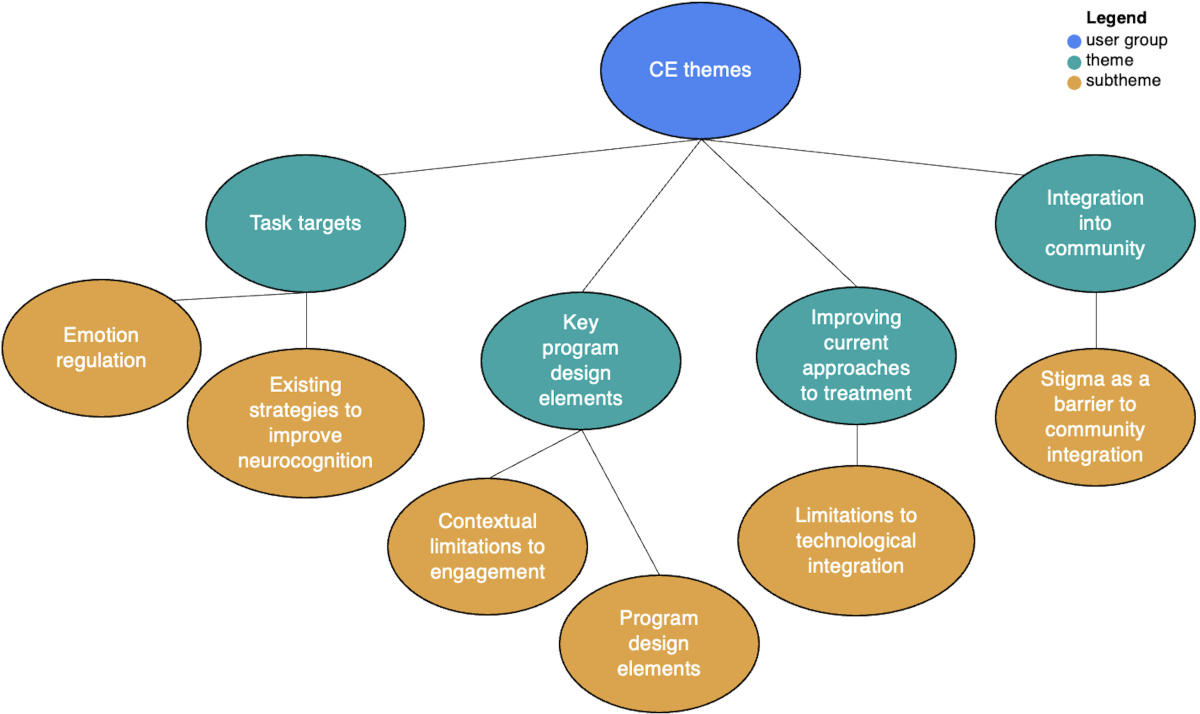
Content expert (CE) thematic map.

The thematic analysis identified 4 themes important for CEs as follows: (1) the need for a program targeting cognitive functioning (task targets), (2) key program design elements, (3) improving current treatment approaches to increase efficacy, and (4) supporting community integration (Figure 4).

**Figure 5:**
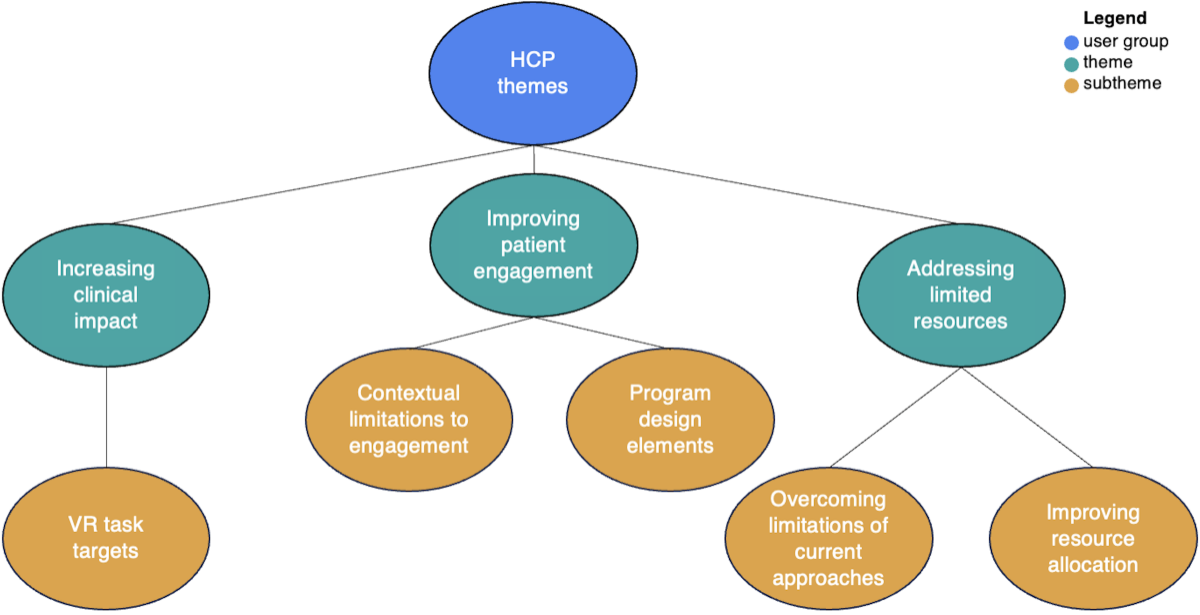
Health care professional (HP) thematic map.

The analysis also revealed 3 themes for HCPs as follows: (1) increasing clinical impact, (2) improving patient engagement, and (3) addressing limited resources (Figure 5). Together, these themes influenced the development of the program, ensuring perspectives and suggestions from the CEs and the HCPs were considered at every step of its creation. Each of these themes and their associated subthemes are elaborated in subsequent sections with selected extracts from the narratives of participants illustrating them (a more comprehensive list of quotes is provided in Multimedia Appendix 2).

### CE Thematic Analysis

#### Theme 1: Cognitive Impairments to Target

##### Overview

One prominent theme that was mentioned during the focus groups was the task targets of an intervention, which include the different neurocognitive and social-cognitive domains in which CEs had difficulties, the strategies they tended to use, and what they would like to work on going forward (Table 1).

CEs tended to tie their neurocognitive challenges, such as planning and attention deficits, to daily life tasks. In 4 of the 9 working groups, CEs also reported memory difficulties, such as remembering to eat or remembering the names of people. In addition, in 4 working groups, CEs discussed social cognition challenges, such as trouble understanding why an acquaintance stopped acting friendly toward them despite there being no overt change in their past interactions. Others expressed difficulties communicating with others and regulating their emotions in stressful situations.

**Table 1:** Content experts’ cognitive impairments to target quote exemplars.

Theme			Quotes
Cognitive impairments to target			“I have difficulty with traveling [and] forgetting when to get off the bus.” “I was going to this gym, and I would say bye to the reception woman, and one day I asked about some workout tips, and she stopped saying bye and I tried to understand why she stopped, and I exhausted myself trying to understand.”
**Subthemes**			
	Impact of emotions and stress on cognition	“The moment that my emotional state is not as well as it should be, like if you’re not calm and capable for doing very well and performing the best, then I cannot remember nothing.” “I have this assignment that was due within a month, and it was for my course, and I set myself up earlier, I gave myself three weeks and started working on it even an hour a day but by the final week I stopped sleeping well and I wasn’t able to focus.”	
	Existing strategies to improve cognition	“I write everything down. I note everything. I don’t do anything with memory.” “The way that I used my phone at least I set up reminders so that I get a weekly reminder, two-day reminder, and then the day of the event.”	

##### Subtheme: Impact of Emotions and Stress on Cognition

CEs explicitly mentioned how their emotional state or environment (eg, the time of day and social context) influenced their neurocognitive and social-cognitive functioning. One CE shared that their memory tended to be poor during social situations, thus affecting their ability to remember faces and names. Another CE shared that their short-term memory was often influenced by their emotional state and affected their level of functioning at work which in turn required them to rely more on compensatory strategies, such as asking their manager to write down their tasks.

##### Subtheme: Existing Strategies to Improve Cognition

For neurocognitive deficits, CEs highlighted their use of mobile phones and note-taking. CEs equally discussed the benefits of using technology (eg, using the reminder function on their phone) versus more traditional external memory aids (eg, a paper agenda). A few CEs focused more on behavioral or environmental modifications to help reinforce memory. One CE even mentioned they wanted to maintain their current level of memory performance and exclusively used their phone to write down appointments. In addition to memory aids, one CE mentioned they try not to overcomplicate their life to avoid stress as it could affect their cognitive abilities. Engaging in social interaction and asking friends for help was also repetitively suggested as a helpful tool. Moreover, 2 CEs shared that they could complete tasks, such as writing an article or setting up a “game night” if they planned ahead. Aside from these strategies, CEs also mentioned the importance of psychoeducation in managing their difficulties better and suggested implementing it in VR.

Finally, CEs shared social cognition strategies in 3 working groups, although fewer strategies were discussed compared to the conversations about existing strategies to support neurocognition. One suggestion was to ask individuals how they were feeling and be more direct when interpreting the emotions of another individual. Other CEs had tips for reducing stress, which could ultimately help manage their cognitive difficulties.

#### Theme 2: Key Program Design Elements

CEs highlighted several key program design elements of an intervention to support rehabilitation. For example, participants expressed:

It’s not a matter of harder or easier. It matters to be realistic and not complicated.

If you get off the bus or walk or you can choose to walk, if you have a time restriction you can still choose to walk or take the bus and then go grocery shopping for a list of things and manage the money...I just find it important to be able to make these things grounded in the reality context.

Notably, CEs identified the importance of an immersive and interactive program environment across 5 working groups. One CE highlighted that the immersive aspect was more important than the difficulty of the program due to the importance of translating the skills learned in the session to daily life. While CEs emphasized the importance of an immersive program, there was debate about the impact of multiple-choice questions on specific tasks on program immersion. One CE expressed that they liked this option as it allows users to learn from different approaches and see how choices affect later actions. Other CEs voiced that the multiple-choice questions could break the VR immersion.

CEs also discussed the importance and need of a program that promotes the translation of skills and strategies learned in the session to daily life. One suggestion to promote translation was to design tasks reminiscent of those CEs encountered in their daily life. Another way to promote translation into reality was to incorporate environmental elements into the program. For example, one CE proposed that if a person forgot their destination while traveling on a bus, they could ask the driver for directions.

Two program design elements were also briefly discussed by the CEs, including having avatars from diverse cultural backgrounds and the ability to change the difficulty level of a program.

#### Theme 3: Improving Current Approaches to Treatment

##### Overview

CEs discussed the impact of pharmacological and psychotherapy interventions and voiced some of their limitations. Such limitations included the negative impact of medication on their cognition and the sparsity of programs available:

Especially for like thinking skills like memory attention and executive function is not something that you live with. It is not related with other symptoms, so we need those kinds of therapy.

Taking medicine, it kind of dulls the mind.

CEs conversed about integrating technology into cognitive rehabilitation approaches to increase treatment efficacy in 4 working groups, which was based on their prior experiences of phones as supportive devices. CEs shared how they relied on their phones to remember appointments; some participants identified a higher chance of forgetting an appointment if it was not entered into their calendars when booked. Interestingly, CEs expressed greater emphasis on the role of technology in supporting social cognition development (rather than neurocognition development), where technology could enable users to practice how to interact in social interactions.

##### Subtheme: Limitations to Technological Integration

While CEs supported technology integration into treatment interventions, they also highlighted concerns:

Not everyone can get data on their phone or has access to WIFI.

When do you start introducing a back-and-forth aspect to a simulation? It opens the door for more back and forth just to keep things from getting confusing, and that might cause some issues later down the line for the programming.

One such concern was that individuals may not have remote access to internet and mobile data, thus affecting the accessibility of internet-dependent interventions delivered through phones. In addition, CEs emphasized 2 ways in which VR interventions are impacted by its equipment. First, VR interventions are constrained by computers that meet specific hardware requirements and are likely more difficult to access. Second, unfamiliar technology could negatively interact with the VR navigation experience; VR controllers differ from typical game controllers. As such, it could take time for a user to become accustomed to the VR interface, potentially affecting the depth of knowledge and strategies learned.

#### Theme 4: Integration Into Community

##### Overview

CEs highlighted their need to socialize and the necessity for better community integration:

I also think that the virtual reality should be fused with the community.

Something that those people, they need drastically. They need something interesting to be involved in their life. They need to socialize, they need to go out.

CEs emphasized the importance of interacting with others to improve their social skills and reduce isolation; a recurring topic was loneliness. While this need was emphasized, some CEs voiced challenges with social interactions, including situations in which individuals wanted to socialize with others but could not start a conversation. Other CEs specifically mentioned their difficulty in talking about themselves and elaborating on their hobbies, which were limited as they mostly spent their time at home “playing games” and “not being able to afford anything.” In addition to this need for social interaction, CEs expressed the importance of aiding disabled individuals with integration into the community. One CE mentioned programs that help disabled individuals integrate within the community and meet others with similar disabilities.

##### Subtheme: Stigma as a Barrier to Community Integration

CEs disclosed how stigma increased the difficulty of integrating within the community and restricted their level of involvement:

I explain to people that I have a long-term disability that could affect my cognition. Everyone has their struggles, and they would understand. I don’t give details that I have schizophrenia.

There is a lot of stigma and discrimination. Maybe they discriminate against you because they think that you are here, and you may be dangerous.

Many CEs shared past experiences of feeling stigmatized by other individuals due to their mental health conditions. One CE expressed that others may perceive them as “dangerous” while another CE shared that they feel like others have preconceptions about their behavior. Considering these experiences, one CE suggested that a program could provide psychoeducation on navigating social situations where users encounter stigma and negative stereotypes being expressed by an avatar. In addition, a program could include a task reflecting the above situation so that users can practice responding.

### HCP Treatment Needs

#### Theme 1: Increasing Clinical Impact

##### Overview

HCPs emphasized a need for more clinically relevant programs targeted toward individuals living with a psychotic disorder:

In my sense this is bridging the gap (in psychiatric care) that we’ve been up against for 20 or 30 years.

I found there is a gap between the psychological and psychiatric world to the actual functioning. I think this would be very great for identifying what specific problem might be because we often are focused on is a task completed or not, but we don’t spend a lot of time discussing partially completed tasks and why the task broke down.

HCPs identified a gap related to translating approaches that support neurocognition and social cognition to psychosocial functioning. Participants conveyed a need for a program that improves neurocognition and social cognition within the context of the functioning of their patients. The use of technology, including VR, to deliver CR could bridge this gap and support the psychosocial functioning of users.

##### Subtheme: Cognitive Impairments to Target

HCPs identified several domains of cognition that a program should target, notably memory and executive functioning:

We’re all clearly craving modules or elements in our clinical practice that help with social cognition? Because the social cognitive landscape for patients is where they grind to a halt.

From my point of view, which is a very practical point of view [is] from discharging people from hospital and can they navigate kind of basic tasks in the community.

HCPs also described a general need to enhance social cognition in 2 working groups but did not identify specific domains. A main portion of the HCP working groups was spent discussing how a program should promote the transfer of learned skills and strategies to the daily lives of their patients*.* HCPs described the impact of neurocognitive impairment on psychosocial functioning, which could affect their recovery and transition from inpatient to outpatient care.

##### Subtheme: Generalizability of the Program

Some HCPs expressed that a program should be generalizable to the clinical variations seen in psychotic disorders. For instance, a CR program in VR should consider the difference between active and chronic-stage psychosis. HCPs also mentioned that when treating a psychotic disorder population, comorbid conditions (eg, sleep apnea) should also be considered. Similarly, 2 HCPs also highlighted the generalizability of a program to other populations marked by cognitive impairment. There was discussion among the HCPs about balancing the broad applicability of the program with ensuring that the tasks target neurocognition, social cognition, and psychosocial functioning. One HCP noted that if the program tasks were too broad, the program may not adequately meet the needs of the users. While the clinical significance of treatment is greater when it is generalizable, future research is also required to investigate whether these broad VR tasks adequately address the needs of users.

#### Theme 2: Improving Patient Engagement

##### Overview

The second theme reflected a need to improve patient engagement in treatment approaches (Table 2). HCPs expressed a need for more patient engagement in the services offered by their clinics and, more broadly, on an institutional level. Motivation and contextual limitations were identified by HCPs as critical factors that influence treatment engagement.

**Table 2:** Health professionals’ improving patient engagement quote exemplars.

Theme			Quotes
Improving patient engagement			“It is the same patients showing up for everything sometimes. We have full groups but it is the same 50 people doing all the groups. We have 1500 program patients, but really over 1000 of them the physician knows. Less than 500 are outpatients taking part in the program. And I don’t think the 1000 patients not taking part in the program aren’t doing so because they’re doing so well.”
**Subthemes**			
	Contextual limitations to engagement	“The question becomes, we would love to be spending more time on cognition but there is nasalism there, and we run into the problem of risk management, safety, medication, morbidity, illness morbidity, family needs, housing needs, issues of poverty.” “We have to be mindful that our medications that we prescribe are not pro-cognitive, they are de-cognitive, and it has changed the prescription culture that we prescribe medication that clearly deteriorates our mission.”	
	Program design elements	“I think finding a good land, or thinking about personalized medicine, or patient preferences is really important.” “We have a little more resource but we are also quite limited in terms of any real cognitive remediation. We can do a baseline neurocognitive assessment on almost everybody in this clinic but then from there, there is one kind of cognitive remediation group.”	

##### Subtheme: Contextual Limitations to Engagement

HCPs listed several contextual limitations to engagement, such as lack of time, lack of social support, and socioeconomic background. For instance, their patients typically face challenges to satisfy basic physical needs, which can affect their ability to be physically and mentally present during appointments. HCPs highlighted accessibility as a contextual limitation to engagement where their patients often encounter transportation issues traveling to and from appointments. Some HCPs discussed how medication side effects may negatively impact neurocognition. They also emphasized a lack of pharmacological approaches available to enhance neurocognition. Regarding the program specifically, HCPs voiced that a prior negative VR experience may affect patient engagement; as such, a VR program should start with a practice portion to reduce the chance of cybersickness and increase the amount of attention directed to the task.

##### Subtheme: Program Design Elements

Across all 3 working groups, HCPs emphasized specific program design elements that could be incorporated into a program to improve engagement, such as the ability to personalize a program to a patient. HCPs voiced that currently available cognitive rehabilitation programs lack the ability to tailor the program to the patient and their goals.

HCPs identified 2 ways a program could provide a personalized approach. First, HCPs mentioned how different approaches to CR may better suit certain patients, especially when considering the chronicity of their condition. The second method to provide a personalized approach was to change various program settings, such as the loudness of distractions. By changing various program design settings, HCPs thought it would enable them to tailor the program to the level of cognitive functioning of their patients. In addition, HCPs expressed that providing a personalized approach would account for the heterogeneous presentation of psychotic disorders and comorbid conditions. For example, HCPs described how a VR program should consider the difference between active and chronic-stage psychosis, as the treatment approach would differ. Two HCPs also highlighted the generalizability of a program to other populations marked by cognitive impairment, such as mood disorders.

Finally, another integral program design element highlighted by HCPs was integrating diversity and inclusivity into the program. HCPs discussed how background avatars could be changed to reflect a greater inclusivity of racial backgrounds and how users themselves could change the coach avatar.

#### Theme 3: Addressing Limited Resources

##### Overview

A prominent theme throughout the HCP working groups was the limited resources available in health care and the need for a program to be effectively incorporated into a health care setting to mitigate these resource constraints (Table 3). HCPs also highlighted current limitations in the diagnosis and treatment of psychotic disorders. Despite this negative lens, they also had suggestions on how to use current resources effectively.

HCPs described what resources needed to be improved in their fields, including a lack of staff and availability. During the working group, HCPs mentioned being “stuck in terms of ... time.” HCPs mentioned that, despite their desire to try new approaches, they do not have the necessary staff to accommodate the substantial number of patients on their waitlists. In turn, this creates an uneven patient-to-health care provider ratio. In addition to staff shortages, patients are subject to long wait times due to, among other things, a long and arduous referral process. The terms “expensive” and “cost” were also used a few times during these discussions.

**Table 3:** Health professionals addressing limited resources quote exemplars.

Theme		Quotes
Addressing limited resources		“[There is] a massive drop off in resources. We have 1500 outpatients in the program. We have 1.4 OTs. We have two psychologists [...], not just [for] our program, and we have 3.5 social workers and 3.5 nurses for our program.” “We have patients ready to go now and we submit a referral, and they have a wait and the waiting kills.”
**Subthemes**		
	Improving resource allocation	“I could see this becoming part of OT. Like we see someone who is referred, and it is almost a year sometimes to see them so how can us as OTs get this going from the get-go?” “I like the idea of thinking about potential of wait room intervention and how relatively brief interventions can be done while a patient is waiting to see the psychiatrist, nurse or social worker.”
	Overcoming limitations of current approaches	“In a clinical interview, our patients usually over endorse their abilities...But looking at that functional endpoint and exploring how the person is actually performing in the task has always been the deficit that we’ve had clinically.” “The nasalism around how fixed are these cognitive deficits, particularly because it looks like most of these cognitive deficits occur before the first episode of early psychosis. So, we’re showing up at the fire after the house has already burnt down.”

##### Subtheme: Improving Resource Allocation

Despite the limited resources, HCPs presented strategies to allocate available resources more efficiently and effectively. In response to staffing challenges, the HCPs suggested delegating interventions, including VR training, directly to allied HCPs, such as OTs. Several HCPs in the working groups felt that a more interdisciplinary and holistic health care method would benefit patients more and create shorter wait times. They also suggested using clinic and hospital waiting rooms to dole out brief interventions and reduce the need for multiple staff while “multipy[ing] your number of clients.” Some HCPs proposed that patients could complete a VR program in a waiting-room setting or while waiting to access a service (referral or waitlist). In these settings, patients could start building on the skills and strategies they would later elaborate on with an OT. For instance, one HCP expressed an interest in patients engaging in a public transportation-orientated module where they initially learn strategies for navigating public transportation before they go out in the community and practice with an OT. In response to the waiting-room intervention idea, HCPs raised the importance of embedding research and other multidisciplinary services (eg, OTs) into a single clinic.

The keywords “quick,” ”brief,” and “immediately” were mentioned a few times during discussions. HCPs emphasized the need for quicker and easier measures, a speedier referral process, and brief but effective intervention methods, such as using waiting rooms to administer interventions.

##### Subtheme: Overcoming Limitations of Current Approaches

In addition to improving resource allocation, limitations in current approaches were brought up. One of the limitations of assessing neurocognition in psychotic disorders is the discrepancy often found between subjective and objective neurocognition. HCPs discussed how patients may overestimate their cognitive abilities, especially if they perform relatively well on a simple cognitive test; however, this test may not accurately measure cognitive difficulties in daily life tasks. Coupled with the need to measure neurocognition accurately, HCPs discussed this need in the context of VR. While it was emphasized that the objective of this program was for training purposes, HCPs were excited about the prospect of VR assessments for neurocognition and community functioning. HCPs were also concerned about whether training cognition is advantageous given the challenges to the schizophrenia algorithm. As cognition is often visibly affected before the first onset of psychosis, HCPs wondered whether it is possible to train cognition to a premorbid state or whether such interventions are fruitless. Therefore, there is a need to define cognitive difficulties in psychosis better to determine which interventions offer more meaningful outcomes.

#### Resulting CR Program: ThinkTactic VR

##### Overview

The needs of CEs and HCPs were placed at the center of the development process, where the VR environment and avatar design, tasks, and scripts were built based on the feedback provided by CEs and HCPs (Textbox 1). For instance, the inclusion of an avatar that guides a user through the program and delivers CR strategies (ie, the VR coach) was recommended by the CEs. On the basis of this feedback, research staff collaborated with the software developers in developing the initial prototype of the VR coach and presented videos of the VR coach in the subsequent working group. The iterative development approach of the entire program, including the VR coach, ensures that the program is continuously meeting the needs of CEs and HCPs. CEs and HCPs treatment needs and suggestions were incorporated to the best of our ability in the iterative program development process to create the final program: ThinkTactic VR.

**Figure 6:**
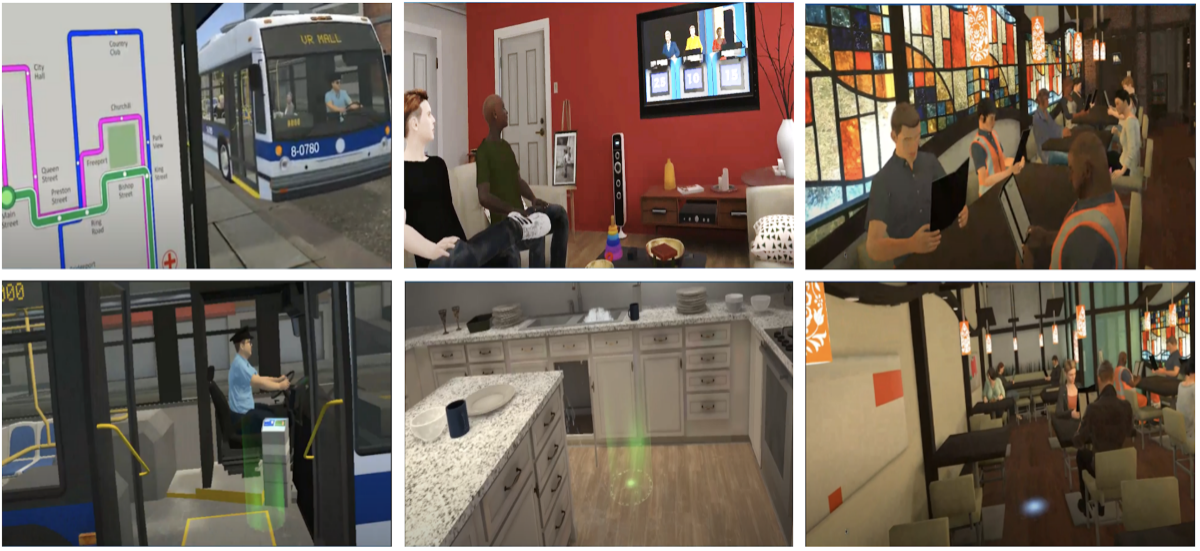
ThinkTactic VR program photos. The city navigation module is featured on the left, the apartment module in the middle, and the restaurant module on the right.

The final CR program in VR includes 3 environments (ie, modules): city navigation, apartment, and restaurant (Figure 6). Each module is composed of 4 levels where the first level serves as an introduction to how to navigate the environment and general tasks. As users progress through the module levels, environmental distractions (eg, ambient noise) and task complexity increase. These neurocognitive and social-cognitive tasks are contextualized in situations an individual is likely to encounter in their life. If a user does not provide the intended answer during a task, they have the opportunity to repeat the task and practice CR strategies. These CR strategies are provided by a VR coach avatar integrated into the module. Users receive instant feedback from the VR coach after responding to a task. If a user is experiencing difficulty, the VR coach will provide CR strategies before the user reattempts the task (thus reinforcing the concept of errorless learning). If a user responds correctly to a task, the VR coach will reinforce CR strategies. This allows users to have repeated exposure and opportunities to practice CR strategies in cognitively challenging real-life scenarios in immersive environments. To support the transfer of learned skills and CR strategies to community functioning, ThinkTactic VR was designed to be accompanied by a trained therapist who also concludes each session with a bridging discussion.

**Examples of user input integrated into ThinkTactic VR and noted for future iterations.**

**Incorporated into ThinkTactic VR**

- creating an interactive and realistic environment
- inclusion of a virtual reality (VR) coach
- inclusion of memory aid
- psychoeducation on selecting balanced meals when heating up food in the apartment module
- selection of the modules
- slower rate of speech from avatars and the VR coach
- tasks targeting neurocognition and social cognition simultaneously
- cultural and gender diversity of avatars

**To be considered in future iterations of ThinkTactic VR**

- ability to customize the VR coach
- adding more tasks to the program
- cultural and gender diversity of the VR coach
- grocery store scenarios involving navigating around the store and calculating prices
- purchasing an item from a store to receive change for a bus trip
- responding to a scenario that involves interacting with the police
- responding to a situation involving stigma

##### VR Module Descriptions

In the city navigation module, the user navigates a virtual city using a public transportation system. The user is tasked to select a bus route that will take them to their destination in the shortest time and least number of stops (Table 4). After selecting the correct bus route, the user boards a virtual bus and must disembark it at the correct bus stop. Later levels build on this task to increase complexity, such as increasing the ambient noise and navigating a detour. In the final level, the user must stop at a grocery store along their route to retrieve bread and a prepurchased order. The second module is situated in a home-based environment where users encounter situations, such as introducing oneself to a houseguest and responding to a flood in a kitchen. Finally, in the third environment, the user takes on the role of a server in a restaurant and is responsible for relaying food orders from customers to the kitchen. In later levels, the number of food items the user needs to remember increases. In all modules, there are 4 levels that feature tasks of increasing cognitive difficulty to increase psychosocial functioning.

**Table 4:** ThinkTactic VR example tasks.

Level			Task description		Example target domains
**City transportation module**					
	Level 2	Selecting a bus route that brings a user to their destination in the shortest amount of time and stops		Problem-solving, decision-making, and attention	
	Level 3	Virtual avatars stare at the user as they board the bus		Emotion regulation, attribution bias, theory of mind, and attention	
	Level 4	Remembering the order number of a grocery order at a store as part of an errand when navigating to the final destination		Memory	
**Apartment module**					
	Level 2	Responding to a flood in the kitchen		Problem-solving, emotion regulation, and attention	
	Level 3	Introducing oneself to an avatar		Emotion regulation and theory of mind	
	Level 4	Selecting takeout food from a series of options while keeping on budget		Problem-solving and decision-making	
**Restaurant module**					
	Level 1 to level 4	Recalling a food order from a table		Memory and attention	

## Discussion

### Principal Findings

The ThinkTactic VR program was codeveloped with CEs and HCPs through an iterative process. Both groups identified treatment needs and provided feedback, which directly informed the design of the program. Their input also highlighted the potential of VR interventions to facilitate transfer, generalizability, and engagement, reinforcing the value of integrating immersive technology into CR. Both groups identified the need for a program addressing neurocognitive and social-cognitive deficits, highlighting the importance of real-world applicability. Each group also brought unique priorities: CEs mentioned the need for an immersive experience, while HCPs focused on tailoring the program to individual clients. HCPs also noted the importance of bridging the research-to-practice gap, improving patient engagement, and overcoming limited health care resources. CEs stressed the need for innovative treatments that integrate technology to enhance efficacy and support community integration.

Our results aligned with previous studies that involved user input during the development of CR programs in VR and identified similar needs, particularly regarding the importance of exercises, feeling immersive and representing real-life situations. Realpe et al [40] adapted an intervention for social cognition in VR with individuals with early-episode psychosis and highlighted the importance for the program to be immersive. Interestingly, Hernandez et al [31] conducted a similar study that analyzed the perspectives of individuals with mood disorders and HCPs while developing a neurocognitive training program. Similar themes were identified, including the importance of an immersive aspect of a VR program, and contextualizing training in real-world scenarios to promote transfer [31]. Subsequently, Hernandez et al [31] modified their prototype to reflect the feedback gathered and the treatment needs of their user groups. ThinkTactic VR is one of the first codeveloped program of CR that targets both neurocognition and social cognition. In addition, rather than modifying an existing VR program, our development approach involved creating a novel CR program in VR from the ground up with input from those with lived experience and HCPs.

ThinkTactic VR was developed according to a framework proposed for the development of VR interventions [22]. As recommended, the development process of ThinkTactic VR integrated empathy into its approaches and promoted inspiration by listening to the needs of CE and HCPs. ThinkTactic VR also featured an extensive collaboration with CEs, HCPs, software developers, and researchers where individuals brainstormed ideas for the program. Any ideas that were not feasible at this point of time due to resource and time constraints were noted down for future refinement of ThinkTactic VR (Textbox 1). Finally, an iterative, bottom-up process was used to design and develop ThinkTactic VR. The iterative approach to program development facilitated continuous user feedback loops that ensured any changes incorporated continued to integrate the needs of CEs and HCPs.

ThinkTactic VR also incorporates the 4 essential components of a CR program identified in a working group [9]. First, a VR coach that provides CR was integrated into the program. The program is also designed for a user to be accompanied by a trained therapist. This therapist facilitates engagement with the program and supports the user in identifying how CR can be applied to their functioning goals. Second, ThinkTactic VR features tasks that train neurocognition and social cognition in the context of cognitively challenging scenarios that resemble those encountered in daily life. Users engage in multiple repetitions of a task throughout 4 levels where the tasks increase in difficulty, providing continuous training. These tasks aid a user in developing problem-solving strategies and promote monitoring strategies, the third component of a CR program. Finally, delivering CR in VR may increase the transfer of learned skills and strategies that ultimately increase psychosocial functioning. Individuals receive more exposure to cognitively challenging tasks they might otherwise avoid. They can also practice multiple times in a safe environment that resembles situations they may encounter in their life, allowing them to apply skills and strategies that facilitate generalization [41].

Initial pilot testing of a ThinkTactic VR module illustrates that it is feasible and acceptable for individuals with psychotic disorders [20]. A pilot randomized controlled trial (NCT05973110) is currently under way to evaluate the initial efficacy, feasibility, and acceptability of the full ThinkTactic VR program. The results of this trial will provide critical insights that will guide further revisions of the program.

### Limitations and Future Directions

While we did our best to incorporate the suggestions of the participants, monetary and time restraints limited the full implementation of all suggestions (Textbox 1). This study was also disrupted by the COVID-19 pandemic. Working groups were initially delivered in person (CE working groups 1 to 5 and HCP working group 1) where participants could directly test the VR program and provide feedback. With the onsite restrictions introduced to reduce the transmission of the COVID-19 infection, all working groups were moved to a web-based format. Instead of immersing the participants in the VR program, participants watched prerecorded videos of the program. Accordingly, there were limited opportunities to test the VR equipment (eg, headset and controllers). This switch to a web-based format may have changed the perceptions of the program, thus impacting on the feedback provided. Finally, the sample size was relatively small, and the attendance of the CE working groups fluctuated over time.

### Conclusions

ThinkTactic VR is the first VR cognitive rehabilitation program targeting both neurocognition and social cognition for individuals with psychotic disorders. It was codeveloped with individuals with lived experience and HCPs through an iterative process, incorporating feedback and suggestions from design to creation. The program was specifically developed to meet the needs of end users. It aims to improve cognition through scenarios grounded in tasks that simulate daily activities, potentially leading to better community functioning. Future studies are needed to assess its efficacy and further refine its development to ensure implementation and scalability.

## Appendix

Multimedia Appendix 1 Content expert demographics.

Multimedia Appendix 2 Thematic analysis quote examples.

## Abbreviations

CE: content expert
CR: cognitive remediation
HCP: health care professional
OT: occupational therapist
ROMHC: Royal Ottawa Mental Health Centre
VR: virtual reality
