# Supplementary material for: Cognitive Remediation for Psychosis in Virtual Reality (ThinkTactic VR): Qualitative, Iterative, and User-Centered Codevelopment Study
Source: JMIR Ment Health. 2025 Jul 11;12:e69359. doi: 10.2196/69359 (PMC12299945; doi:10.2196/69359)
Supplement: Multimedia Appendix 1 [file mental_v12i1e69359_app1.docx]

**Multimedia Appendix 1**

**CEs Demographic**

| Variable | Statistics | Range |
| --- | --- | --- |
| Years of education^a^, *M* (*SD*) | 16.45 (3.62) | 11-24 |
| Age in years, *M* (*SD*) | 42.55 (12.62) | 21-66 |
| **Sex, *n* (%)** |  |  |
| Female | 3 (27.27) | N/A |
| Male | 8 (72.73) | N/A |
| **Employment status, *n* (%)** |  |  |
| Student | 2 (18.18) | N/A |
| Full-time employed | 1 (9.09) | N/A |
| Unemployed, seeking work | 1 (9.09) | N/A |
| Retired | 1 (9.09) | N/A |
| Unemployed, disability | 6 (54.55) | N/A |
| **Marital status, *n* (%)** |  |  |
| Single | 8 (72.73) | N/A |
| Separated or divorced | 1 (9.09) | N/A |
| Married or common law | 2 (18.18) | N/A |
| Cybersickness, *M* (*SD*) | 16.73 (6.02) | 0-24 |

^a^Years of education reflect high school and post-secondary education
