# Supplementary material for: Cognitive Remediation for Psychosis in Virtual Reality (ThinkTactic VR): Qualitative, Iterative, and User-Centered Codevelopment Study
Source: JMIR Ment Health. 2025 Jul 11;12:e69359. doi: 10.2196/69359 (PMC12299945; doi:10.2196/69359)
Supplement: Multimedia Appendix 2 [file mental_v12i1e69359_app2.docx]

**Multimedia Appendix 2**

**Thematic Analysis Quote Exemplars**

**Content Experts (CEs) Thematic Analysis**

The CEs thematic analysis revealed four treatment needs: the need for a program that addresses neurocognitive and social-cognitive impairments, the key program design elements that should be integrated into a program to support rehabilitation, improving treatment approaches through technology integration, and the need of a program to support community integration.

**Table 1. CE Theme 1: Task Targets**

| Sub-Themes | Examples |
| --- | --- |
| Cognitive difficulties in daily life | - “Sometimes I don’t remember what I ate this morning at all unless I’m hungry, so then I so I try to eat when I’m hungry. But I don’t remember whether I had breakfast or not.” - “I was going to this gym and I would say bye to the reception woman and one day I asked about some workout tips and she stopped saying bye and I tried to understand why she stopped and I exhausted myself trying to understand” - “I have difficulty with travelling, forgetting when to get off the bus. I know how to go there but I have difficulties along the way and I encounter obstacles.” |
| Sub-Themes | |
| Existing strategies to improve cognition | - “I write everything down. I note everything. I don’t do anything with memory. And I feel like that helps with stress.” - “I think the way that I used my phone at least I set up reminders so that I get a weekly reminder, two day reminder, and then the day of the event. So that in itself reinforces the memory ahead. So like one week from now you have… So by hearing it twice is kind of solidifies it even further in my memory, my long-term memory. so it’s like you already know this, and then you hear another reminder, it’s like further reminding you.” |
| Impact of emotions and stress on cognition | - “Short-term memory to me, it depends on my emotional state. Like how I feel, like if you’re happy, if you like the person, if it is important to you, if it is interesting to you, like you have the same interest then I don’t have any trouble, like to remember names, like everything. But the moment that my emotional state is not as well as it should be, like if you’re not calm and capable for doing very well and performing the best, then I cannot remember nothing, like nothing. Like when I work I ask, because you’re stressed out, they give you a new task, and then I’m stressed out and I don’t remember anything even when I know it so well.” - “I have this assignment that was due within a month and it was for my course and I set myself up earlier, I gave myself three weeks and started working on it even an hour a day but by the final week I stopped sleeping well and I wasn’t able to focus or anything by then I just I was unable to like really finish the assignment very well.” - “I feel like I need to train every environment so over time if I’m in the specific environment, I’ll develop copings tragedies to be there and then probably be more comfortable.” |

**Table 2. CE Theme 2: Key Program Design Elements**

| Sub-Themes | Examples |
| --- | --- |
| General theme quotes | - “It’s not a matter of harder or easier. It matters to be realistic and not complicated. Like the daily basis is what is you know, the real life is actually the best teacher. Like if because the units make the situation complicated or different depends you know so actually like when you start applying to people, when they start participating, and when you use it with them…” - “I would like to see diversity in terms of culture, it would good to see different types of people in VR environment.” - “If you get off the bus or walk or you can choose to walk, if you have a time restriction you can still choose to walk or take the bus and then go grocery shopping for a list of things and manage the money…I just find it important to be able to make these things grounded in the reality context.” |

**Table 3. CE Theme 3: Improving Current Approaches to Treatment**

| Sub-Themes | Examples |
| --- | --- |
| General theme quotes | - “Well cognitive behavioural therapy. I learned the catch it, check it and change it. Specially for like thinking skills like memory attention and executive function is not something that you live with. It is not related with other symptoms so we need those kinds of therapy.” - “If it's not on my phone, then I don't remember it. Like because people always offer you these cards with appointments on it. Like at the hairdressers, she would want me to write out on a card but no I'm gonna put on my phone right now otherwise this paper just goes missing. And even with the doctors appointments here, like I find that they give you a card and like no just tell me the time and date and I'll just put it in my phone right now and then I'll remember it because otherwise with the cards, it’s not in my calendar and I completely forget that appointment. - “For appointments and day-to-day activities. Like even I don't remember the week or day.” - I started and I went to places but when it comes to seeing new people, I have a hard time just starting a conversation so in that case, it is like, I feel like I’m an outsider and I don’t know how to say or how to start. In this context, its very helpful for someone like me or someone people like in my situation. It’s helpful. It’s a good step toward that goal.” |
| Sub-Theme | |
| Limitations to technology integration | - “I’m going to have to throw a wrench in that idea, because not everyone can get data on their phone or has access to WIFI.” - “Well I was saying that the bus could have WIFI and mobile phone, it costs money so let’s pretend you don’t have the money.” - “When you start introducing a back and forth aspect to a simulation? It opens the door for more back and forth just to keep things from getting confusing, and that might actually cause some issues later down the line for the programming like to add more and more dialogue for the interactions.” - “PARTICIPANT 1: Guess you need a pretty powerful computer to run the VR system, right?   SPEAKER 1: Yeah.  PARTICIPANT 1: Because we couldn’t do that on our computer unless we had Alienware.” |

**Table 4. CE Theme 4: Integration into Community**

| Sub-themes | Examples |
| --- | --- |
| General theme quotes | - “My advice is that try to grab a friend like for example I am talking about a good friend, not like not like, you know like a friend, and then like ask you know I'm stuck here. Because usually when you share your problems and basically you will be more motivated. Like not too disappointed at the same time you get help. This will like you know this like, being a human, you're a social animal, you do need to socialize with people.” - “Going out actually, you have a different environment. Not only you meet people, but to meet people and communicate with them you need social skills.” - “I also think that the virtual reality should be fused with community.” - “Something that actually those people, they need drastically. They need something interesting to be involved in their life. They need socializing, they need to go out, they need – because gamer, this is what everyone does. Its something that is very simple to escape loneliness, to escape not being able to afford anything. To escape, your complete out of the reality, sometimes. You know, not having interest of anything.” |
| Sub-Theme | |
| Stigma as a barrier to community integration | - “There is a lot of stigma and discrimination. Maybe they discriminate you because they think that you are here, and you may be dangerous. - “Because I fear that other people won't understand my neuro diversity as to why maybe I walk around and contemplate a bit and see preoccupied, like if somebody sees me kind of like being in my own head too much, they’ll think oh you okay? Is there is something wrong with you?” - “I agree with other people that you should not share too much and be careful with who you share…” - “I explain to people I have long-term disability that could affect my cognition. Everyone has their struggles and they would understand. I don’t give details that I have schizophrenia.” - “Creating reactions of people with stigma and knowing how to react. Someone who judges you for no reasons. And having the right reactions.” |

**HP Thematic Analysis Quotes**

Three themes emerged from the HPs thematic analysis: increasing clinical impact, improving patient engagement in treatment, and addressing the limited resources available in the healthcare system.

**Table 5**. **HP Theme 1: Increasing Clinical Impact**

| Sub-Theme | Examples |
| --- | --- |
| General theme quotes | - “I found the gap between the psychological and psychiatric world to the actual functioning executive function cognitive world, I find there’s a bit of a gap there. I think this would be very great for identifying what specific problem might be because we often are focused on is a task completed or not, but we don’t spend a lot of time discussing partially completed tasks and why the task broke down. I think this would be an excellent tool for identifying those sorts of intermediate outcomes.” - “In my sense this is bridging the gap that we’ve been up against for 20 or 30 years.” |
| Sub-Themes | |
| Task targets | - “I would say also I think also one of the most particularly executive dysfunction and frontal lobe function is the thing that I hold the most up for.” - “I mean we're all clearly craving modules or elements in our clinical practice that help with social cognition, right? Because the social cognitive landscape for patients is where they grind to a halt, right?” - “One of our challenges is to determine, with the help of occupational therapy is, are they safe, can we say whether they can cognitively manage being discharged. And usually this task revolves around can they adequately get food for themselves and navigating this, and can they navigate adequately around the city, and even this is massively challenging. And I get lots of calls from patients that have got lost in the bus system and can’t figure out how to get here even though they've been shown once or twice before. Can they safely operate a kitchen so that they're not leaving the stove on and not burning down their apartment because that's a massive issue and can they pay bills so they're not getting evicted?” - “From my point of view, which is a very practical point of view from discharging people from hospital and can they navigate kind of basic tasks in the community either kind of targeting the basic tasks that were kind of looking for so that being the primary starting point, it's highly relevant to a lot of the people that I'm working with.” |
| Generalizability of the program | - “I think a lot of that in terms of a clinical staging approach, maybe people who are in that early psychosis stage, or people who have moderate but not severe cognitive deficits, without a lot of high negative symptoms later in the illness, might be really good candidates for a restorative approach, and those who have support members or a caregiver, they may not even need extensive cognitive remediation but rather cognitive stimulation is a good starting point for them. Whereas really high negative symptoms, those with severe and long-standing cognitive deficits who haven’t worked, socialized, recreated in 15 years, we may be able to find a large effect size in some measures, but the degree they have to climb over the course of their illness means they are still somewhat impaired, and we may look to some of those compensatory approaches.” - “I am trying more and more to get out in the community in their environment, and then it is very individualized too, so it is harder to run a group when it is not applicable to a bunch of different clients.” |

**Table 6. HP Theme 2: Improving Patient Engagement**

| Sub-Theme | Examples |
| --- | --- |
| General theme quotes | - “I think it becomes almost, that it is the same patients showing up for everything sometimes. So we have full groups but it is the same 50 people doing all of the groups. So we have 1500 program patients, but really over 1000 of them the physician knows. Less than 500 are outpatients taking part in the program. And I don’t think the 1000 patients not taking part in the program aren’t doing so because they’re doing so well. I think it is the opposite.” |
| Sub-Themes | |
| Contextual limitations to engagement | - “The question becomes, we would love to be spending more time on cognition but there is nasalism there, and we run into the problem of risk management, safety, medication, morbidity, illness morbidity, family needs, housing needs, issues of poverty, so this is what is the core of my illness, but I am unable to focus on it because all of my energy has absorbed in all the other things.” - “And I see it as a vicious cycle too. Because a lot of our patients are not performing well enough to manage their well being. And they don’t have access or energy to come and focus on their health. - “A lot of our patients rely on us to give them an in or they can’t afford the bus fares even to get in and out of appointments.” - “We have to be mindful that our medications that we prescribe are not pro-cognitive, they are de-cognitive, and it has changed the prescription culture that we prescribe medication that clearly deteriorates our mission is something we can do now.” - “I think you're right with that. I think you're going to have individuals who may benefit with this, but who may need a significant amount of time just to be in that virtual reality experience and get comfortable within that experience because it may be unsettling for them for a variety of different reasons, and even just to be able to figure out how to move and how to pick things and how to make those selections which are necessary to them to be able to function in more complex tasks..” |
| Program design elements | - “We are also quite limited in terms of any real cognitive remediation. We can do a baseline neurocognitive assessment on almost everybody in this clinic but then from there, there is one kind of cognitive remediation group that they can give them which is more focused on skills training, techniques to enhance their performance. - “I think finding a good land, or thinking about personalized medicine, or patient preferences is really important. There are probably a lot of people who would not want to do cognitive remediation in the sense of a restorative approach or an enhancement approach but would be not only preferred but good candidates for a compensatory approach.” - “So, there could there be the option though if they do the more complex one that then you tell them we're going to add like auditory stimulation just so they're more aware they can maybe practice without it and then slowly add it on top, instead of all at once.” - “I don’t think we’re there yet from a technology perspective, but I know everything that goes through my head right now is through that lens of “what are we actually doing to address these cultural stereotypes and boundaries when they come for therapy and treatments?” - “It would be nice if people could see themselves in the avatars.” |

**Table 7**. **HP Theme 3: Addressing Limited Resources**

| Sub-Theme | Examples |
| --- | --- |
| General theme quotes | - “[There is] a massive drop off in resources. We have 1500 outpatients in the program. We have 1.4 OTs. We have two psychologists […], not just [for] our program, and we have 3.5 social workers and 3.5 nurses for our program, so some of these can’t service.” - “I am unable to focus on it because all of my energy has absorbed in all the other things, and plus, all of these things are very time consuming and somewhat expensive.” - “We have patients ready to go now and we submit a referral, and they have a wait and the waiting kills. And if a person presents with the motivation to take part in something, we need to provide it immediately.” - “[…] working with people with psychosis and schizoaffective disorders for 5-10 years or longer, often we have to have their intervention for much shorter chunks and have them come back or have multiple small assessments with the neuropsychologist and multiple small assessments. Whereas we can get away with less frequent and longer interventions with early psychosis. So that also just a time suck on staff in terms of having them hands on with people there.” |
| Sub-Themes | |
| Improving resource allocation | - “This is one of the things we are trying to do this at our intervention at the clinic by creating multidisciplinary problems so that people that once they come in and see the psychiatrist, they get the services from the team immediately, there is no waiting around for a few weeks or months to start the intervention.” - “That is not a way that I thought before and until I started working close with OTs.” - “I could see an occupational therapist doing it, and I could see this becoming part of OT. Like we see someone who is referred and it is almost a year sometimes to see them so how can us as OTs get this going from the get-go?” - “I think everything needs to be embedded in the clinic setting. So if we are doing intake, we need to say okay you are seeing me today, the psychologist, the social worker, this is what you do, this is where you go this is part of our program.” - “I like the idea of thinking about potential of wait room intervention and how relatively brief interventions can be done while a patient is waiting to see the psychiatrist, nurse or social worker, I think there is some lost opportunity to try to do some more independent training in that environment.” |
| Overcoming limitations of current approaches | - “In a clinical interview, our patients usually over endorse their abilities, right, because the tests are relatively simple and I don’t know if it’s an embarrassment or lack of awareness or actually not having any actual insight in terms of how the visibility is. But looking at that functional endpoint and exploring how the person is actually performing in the task has always been the deficit that we’ve had clinically.” - “The nasalism around how fixed are these cognitive deficits, particularly because it looks like most of these cognitive deficits occur before the first episode of early psychosis. So we’re showing up at the fire after the house has already burnt down is what a lot of us are worried about.” - “[…] everyone has their favourite way of doing it but I think from my perspective I am interested [in] remediating all measures that we can do quickly and easily that have a meaningful outcome.” |
